# Comparative efficacy of vancomycin in treating ST5 and ST764 methicillin-resistant *Staphylococcus aureus* infections in adult patients

**DOI:** 10.1128/msphere.00457-23

**Published:** 2023-10-31

**Authors:** Yaxin Fan, Kaiting Zhang, Mengting Chen, Nanyang Li, Xiaofen Liu, Minjie Yang, Xiaoyu Liang, Jufang Wu, Beining Guo, Huajun Zheng, Yongqiang Zhu, Fengying Zhang, Jingqing Hang, Huifang Zhang, Ruilan Wang, Qing Yuan, Xiaolian Song, Shengbin Wu, Bo Shen, Jing Zhang

**Affiliations:** 1 Institute of Antibiotics, Huashan Hospital, Fudan University, Shanghai, China; 2 Key Laboratory of Clinical Pharmacology of Antibiotics, National Population and Family Planning Commission, Shanghai, China; 3 National Clinical Research Center for Aging and Medicine, Huashan Hospital, Fudan University, Shanghai, China; 4 Phase I Clinical Research Center, Huashan Hospital, Fudan University, Shanghai, China; 5 Shanghai-MOST Key Laboratory of Health and Disease Genomics, Chinese National Human Genome Center at Shanghai and Shanghai Institute for Biomedical and Pharmaceutical Technologies, Shanghai, China; 6 Department of Pulmonary Medicine, Shanghai Putuo District People’s Hospital, Shanghai, China; 7 Emergency and Critical Care Department, Shanghai General Hospital, Shanghai Jiao Tong University School of Medicine, Shanghai, China; 8 Department of Respiratory and Critical Care Medicine, Tenth People’s Hospital of Tongji University, Shanghai, China; 9 Department of Nephrology, Shanghai Ninth People’s Hospital, Shanghai, China; Hackensack Meridian Health Center for Discovery and Innovation, Nutley, New Jersey, USA

**Keywords:** ST5, ST764, methicillin-resistant *Staphylococcus aureus*, vancomycin, efficacy

## Abstract

This study compared the impact of sequence type 5 (ST5) and ST764, on the efficacy of vancomycin treatment in methicillin-resistant *Staphylococcus aureus* (MRSA) infections. From July 2012 to June 2020, a prospective observational study was conducted in five hospitals in China, enrolling 90 patients with MRSA infections, including 44 patients with ST5-MRSA and 46 patients with ST764-MRSA infections. All strains were subjected to minimal inhibitory concentration (MIC) determination, biofilm expression, heterogeneous vancomycin-intermediate *S. aureus* (hVISA) screening, and whole-genome sequencing. Vancomycin therapeutic drug monitoring was conducted, and the 24-hour area under the curve (AUC_0-24_) and AUC_0-24_/MIC values were calculated. ST5-MRSA shows elevated hVISA/VISA presence, increased biofilm formation, and higher presence of virulence genes like *tst*, *sec*, and *sel*. ST764-MRSA expresses *seb*, aiding infection in elderly patients in the community. Additionally, patients with ST764 infections exhibit higher vancomycin AUC_0-24_/MIC values, and fewer tracheotomies compared to ST5. Clinical signs and symptoms improvement were observed in 27 (61.4%) and 33 (71.7%) patients in the ST5 and ST764 groups, respectively (*P* = 0.372). On the other hand, 28 (63.6%) and 39 (84.8%) patients had laboratory-confirmed bacterial eradication in two groups, respectively (*P* = 0.029). Multivariate analysis showed that the virulence genes, such as the *tst* gene, were a risk factor for bacterial persistence (adjusted odds ratio, 4.509; 95% confidence interval, 1.216 to 16.724; *P* = 0.024). Our study showed that vancomycin was less effective in treating ST5-MRSA infection compared to ST764-MRSA infection, in part because ST5-MRSA is healthcare-associated MRSA while ST764-MRSA carries genetic characteristics of community-associated MRSA.

**IMPORTANCE:**

Methicillin-resistant *Staphylococcus aureus* (MRSA) is a bacterium that is resistant to multiple drugs and can cause serious infections. In recent years, one of the most widespread strains of MRSA worldwide has been the clonal complex 5 (CC5) type. Sequence type 5 (ST5) and ST764 are two prevalent CC5 strains. Although ST5 and ST764 are genotypically identical, ST764 is classified as a hybrid variant of ST5 with characteristics of community-associated MRSA (CA-MRSA). In contrast to ST5, ST764 lacks the *tst* and *sec* genes but carries the staphylococcal enterotoxin B (*seb*) gene. Vancomycin is commonly used as the first-line treatment for MRSA infections. However, it is currently unclear whether the genetic differences between the ST5 and ST764 strains have any impact on the efficacy of vancomycin in treating MRSA infections. We conducted a prospective observational study comparing the efficacy of vancomycin against ST5-MRSA and ST764-MRSA in five hospitals in China. There were significant differences in bacteriological efficacy between the two groups, with virulence genes, such as the *tst* gene, being a risk factor for bacterial persistence (adjusted odds ratio, 4.509; 95% confidence interval, 1.216 to 16.724; *P* = 0.024). In the future, it may be necessary to consider personalized vancomycin treatment strategies based on the genetic characteristics of MRSA isolates.

## INTRODUCTION

Methicillin-resistant *Staphylococcus aureus* (MRSA) is a multidrug-resistant bacterium that causes serious infections, including bloodstream infections, pneumonia, skin and soft tissue infections, and infective endocarditis, with high morbidity and mortality. Additionally, MRSA can lead to prolonged hospitalization, putting pressure on healthcare systems (1–3).

Among prevalent MRSA strains, the clonal complex 5 (CC5) type is one of the most widely spreading strains worldwide in recent years (4, 5). As the predominant subtype of the CC5, sequence type 5 (ST5) mainly originated from New York/Japan (4) and is currently one of the major MRSA endemic strains in East China (6, 7). The ST5 clone is a prevalent strain associated with nosocomial infections (4) and is characterized by the presence of staphylococcal cassette chromosome *mec* (SCC*mec*) type II and positive for toxin-type toxin 1 (*tst*) and enterotoxin C (s*ec*) genes, as well as resistance to multiple antibiotics (8). ST764 also belongs to the CC5 family and was first identified in Japan. It has since been widely disseminated in both hospital and community settings (8–10). Recent reports have shown an increasing detection rate of ST764 in China, and it is gradually becoming the dominant strain in various clinical infections, surpassing ST5 (11–13). ST764 clone is a variant of the ST5 lineage. Despite being a healthcare-associated MRSA (HA-MRSA) clone, it is a hybrid variant with community-associated MRSA (CA-MRSA) features (8). ST764 has acquired two new mobile genetic elements during evolution: the arginine catabolism mobile element II (ACME-II) and SaPInn54. This acquisition resulted in a virulence profile similar to that of CA-MRSA, as these elements carry genes such as ACME *arcA* and the staphylococcal enterotoxin B gene (*seb*) (8, 14). Notably, ST764-MRSA also exhibited enhanced expression of cytolytic peptide genes. However, it was observed that ST764-MRSA lacked Panton-Valentine leukocidin, a common virulence factor found in CA-MRSA strains.

Vancomycin is the primary treatment option for MRSA infections, given its strong antibacterial activity against this pathogen (15, 16). Despite having similar genotypes, ST5-MRSA and ST764-MRSA differ in their genetic identity. It is unclear whether this difference affects the effectiveness of vancomycin treatment.

The aim of this study is to conduct a comprehensive comparison of the clinical characteristics, vancomycin pharmacokinetic/pharmacodynamic (PK/PD) indices, and strain genotype characteristics of ST5 and ST764 strains isolated from patients with MRSA infections. The findings of this study will provide insights into the potential impact of vancomycin treatment on the clinical and bacterial efficacy of infections caused by these strains.

## RESULTS

### Patient clinical features

**Fig 1:**
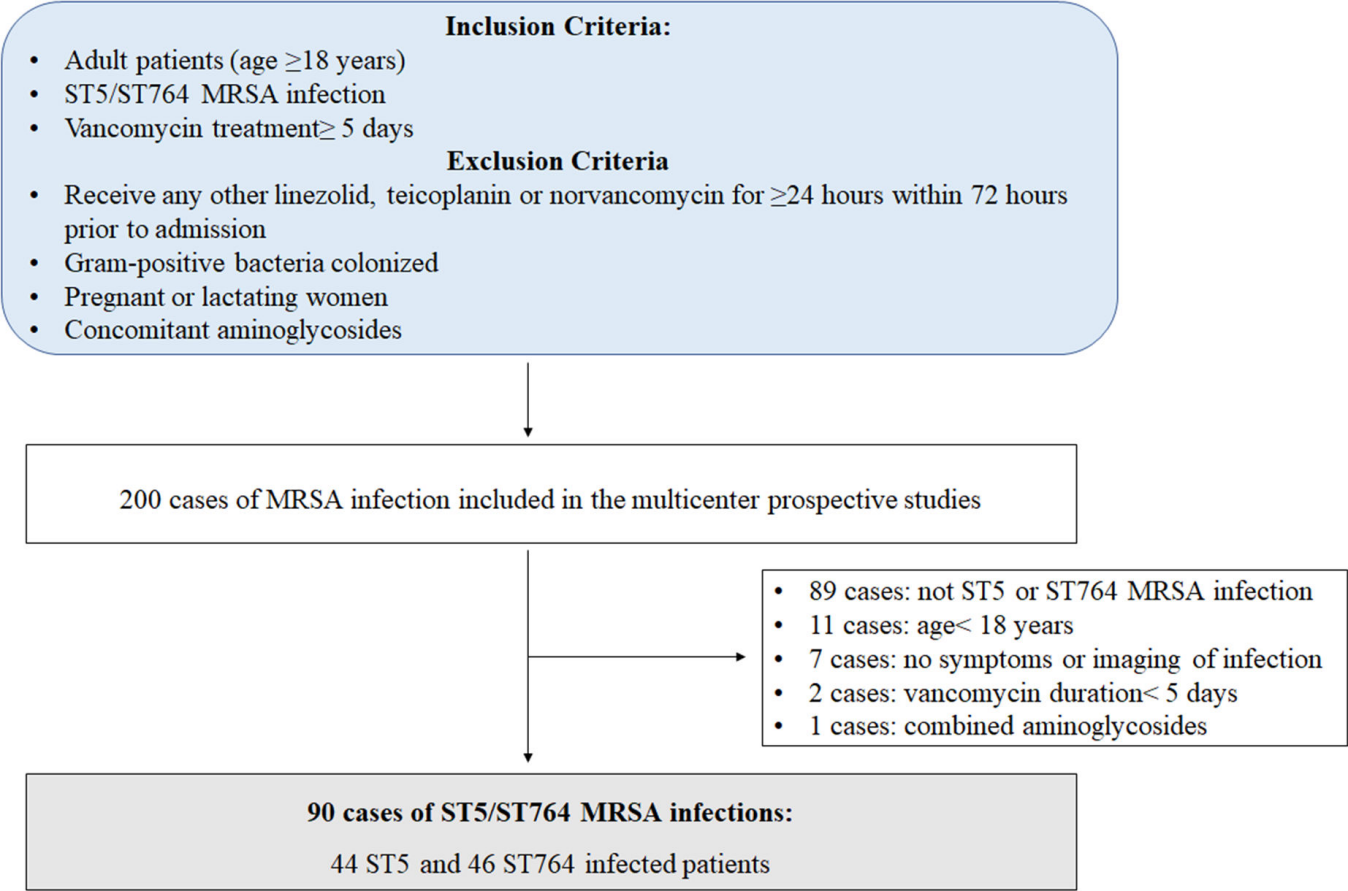
Flowchart of patient enrollment and assessment in this study.

In total, 90 patients with MRSA infections were included in this analysis, 44 (48.9%) patients were identified as ST5-MRSA, and 46 (51.1%) patients were ST764-MRSA (Fig. 1). Clinical features of these diagnosed MRSA-infected patients were thoroughly compared between ST5 and ST764 groups (Table 1). A significant difference in age was observed between the two groups, e.g., patients in the ST764-MRSA group were older than those in the ST5-MRSA group (*P* < 0.001), while no significant differences in gender, weight, body mass index (BMI), admission to the intensive care unit (ICU), or days spent in the ICU were identified. The majority of ST5-MRSA patients (88.6%) originated from tertiary care hospitals, whereas only 26.1% of ST764-MRSA patients exhibited the same origin. In terms of underlying disease, patients in the ST764-MRSA group showed a higher rate of cardiovascular diseases (45.7% vs 22.7%, *P* = 0.027) and strokes (56.5% vs 15.9%, *P* < 0.001). In addition, a higher proportion of patients with ST5-MRSA infection underwent more tracheotomy procedures (43.2% vs 10.9%, *P* = 0.001). Regarding the infection type, pulmonary infections (69 patients, 76.7%) and bloodstream infections (11 patients, 12.2%) accounted for the majority of MRSA infections. ST5-MRSA bloodstream infections were twice as common as ST764, although the difference was not statistically significant (15.9% vs 8.7%, *P* = 0.348).

**TABLE 1:** Demographics and clinical features of infections caused by ST5-MRSA vs ST764-MRSA isolates

Characteristic^*a*^	Total (*n* = 90)	ST5^*b*^ (*n* = 44)	ST764 (*n* = 46)	*P* value^*c*^
Demographic				
Age, years	69 (51, 80)	61 (42, 72)	77 (64, 84)	**<0.001**
Gender, male	63 (70.0%)	29 (65.9%)	34 (73.9%)	0.492
Weight, kg	60 (55, 70)	65 (55, 72)	60 (55, 70)	0.126
BMI, kg/m^2^	22.0 (20.0, 24.0)	22.8 (20.3, 24.0)	21.8 (19.0, 24.0)	0.190
ICU				
ICU admission	53 (58.9%)	22 (50.0%)	31 (67.4%)	0.133
ICU stay days	11(0, 30)	0 (0, 28.5)	18 (0, 31)	0.206
Occurred in tertiary care hospitals	51 (56.7%)	39 (88.6%)	12 (26.1%)	**<0.001**
Underlying disease				
Cardiovascular diseases	31 (34.4%)	10 (22.7%)	21 (45.7%)	**0.027**
Diabetes	14 (15.6%)	5 (11.4%)	9 (19.6%)	0.386
Strokes	33 (36.7%)	7 (15.9%)	26 (56.5%)	**<0.001**
COPD	3 (3.3%)	1 (2.3%)	2 (4.3%)	>0.999
Autoimmune diseases	1 (1.1%)	1 (2.3%)	0 (0%)	0.489
Trauma	11 (12.2%)	3 (6.8%)	8 (17.4%)	0.198
Solid tumor	17 (18.9%)	10 (22.7%)	7 (15.2%)	0.426
Surgery	39 (43.3%)	23 (52.3%)	16 (34.8%)	0.136
Implant				
Venous catheter	64 (71.1%)	29 (65.9%)	35 (76.1%)	0.355
Endotracheal intubation	30 (33.3%)	19 (43.2%)	11 (23.9%)	0.074
Tracheotomy	24 (26.7%)	19 (43.2%)	5 (10.9%)	**0.001**
Urinary catheter	54 (60.0%)	30 (68.2%)	24 (52.2%)	0.137
Drainage tube	22 (24.4%)	11 (25.0%)	11 (23.9%)	>0.999
Infection site				
Bloodstream infection	11 (12.2%)	7 (15.9%)	4 (8.7%)	0.348
Pulmonary infection	69 (76.7%)	32 (72.7%)	37 (80.4%)	0.459
Central nervous system infections	3 (3.3%)	3 (6.8%)	0 (0%)	0.113
Skin and soft tissue infections	5 (5.6%)	4 (9.1%)	1 (2.2%)	0.198
Urinary tract infection	4 (4.4%)	1 (2.3%)	3 (6.5%)	0.617
Other infection	6 (6.7%)	3 (6.8%)	3 (6.5%)	>0.999
Treatment				
Vancomycin daily dose, g	1.8 (1.2, 2.0)	2 (1.6, 2.1)	1.4 (1.0, 2.0)	**<0.001**
Vancomycin duration	13 (10,18)	13 (10,17)	12 (9,20)	0.399
Combined with β-lactams	58 (64.4%)	30 (68.2%)	28 (60.9%)	0.514
Combined with rifampin	7 (7.8%)	3 (6.8%)	4 (8.7%)	>0.999
Combined with quinolones	4 (4.4%)	3 (6.8%)	1 (2.2%)	0.355
Combined with other antibiotics	25 (27.8%)	11 (25.0%)	14 (30.4%)	0.641

^*a*^ ST, sequence type; BMI, body mass index; ICU, intensive care unit; COPD, chronic obstructive pulmonary disease.

^*b*^ Continuous variables were expressed as median (interquartile range) and categorical variables were summarized as the number of cases (%) in the table.

^*c*^ *P* values< 0.05 are shown in bold.

### Vancomycin therapeutic drug monitoring (TDM) and PK/PD analysis

Vancomycin TDM and PK/PD analysis were performed for all patients to enable personalized therapy. As for vancomycin treatment, the daily doses were higher in the ST5-MRSA group than those in the ST764 group (2 g vs 1.4 g, *P* < 0.001), with comparable vancomycin in combination with other antibiotics between the two groups. After the implementation of TDM (Table 2), patients with ST764-MRSA infection had higher trough concentration (*C*_min_) (14.37 mg/L vs 10.09 mg/L, *P* = 0.011) but comparable peak concentration (*C*_max_) compared to ST5. The 24-hour area under the curve (AUC_0-24_) was also higher in ST764 but not significantly different from ST5. Interestingly, the AUC_0-24_-to-minimal inhibitory concentration ratio (AUC_0-24_/MIC) is significantly higher in the ST764 group (732 vs 500, *P* < 0.001).

**TABLE 2:** Correlation between vancomycin pharmacokinetics and pharmacokinetic/pharmacodynamic indices of infections caused by ST5-MRSA vs ST764-MRSA isolates

Characteristic^*a*^	Total (*n* = 90)	ST5^*c*^ (*n* = 44)	ST764 (*n* = 46)	*P* value^*d*^
PK^*b*^				
*C*_min_, mg/L	11.18 (7.53,16.70)	10.09 (5.03,13.72)	14.37 (8.36, 19.61)	**0.011**
*C*_max_, mg/L	26.31 (19.73, 32.34)	25.78 (20.68, 30.02)	27.94 (19.63,34.40)	0.522
AUC_0-24_, mg·h/L	421 (327, 570)	398 (329, 482)	456 (322, 612)	0.338
PK/PD				
AUC_0-24_/MIC	625 (415, 912)	500 (365, 659)	732 (505,1176)	**<0.001**

^*a*^ PK, pharmacokinetic; PK/PD, pharmacokinetic/pharmacodynamic; *C*_min_, trough concentration; *C*_max_, peak concentration; AUC_0-24_, 24-hour area under the curve; AUC_0-24_/MIC, 24-hour area under the curve over minimal inhibitory concentration.

^*b*^ All the PK parameters were calculated as the weighted average values.

^*c*^ Continuous variables were expressed as median (interquartile range) in the table.

^*d*^ *P* values< 0.05 are shown in bold.

### Phenotypes and molecular characterization of MRSA strains

**Fig 2:**
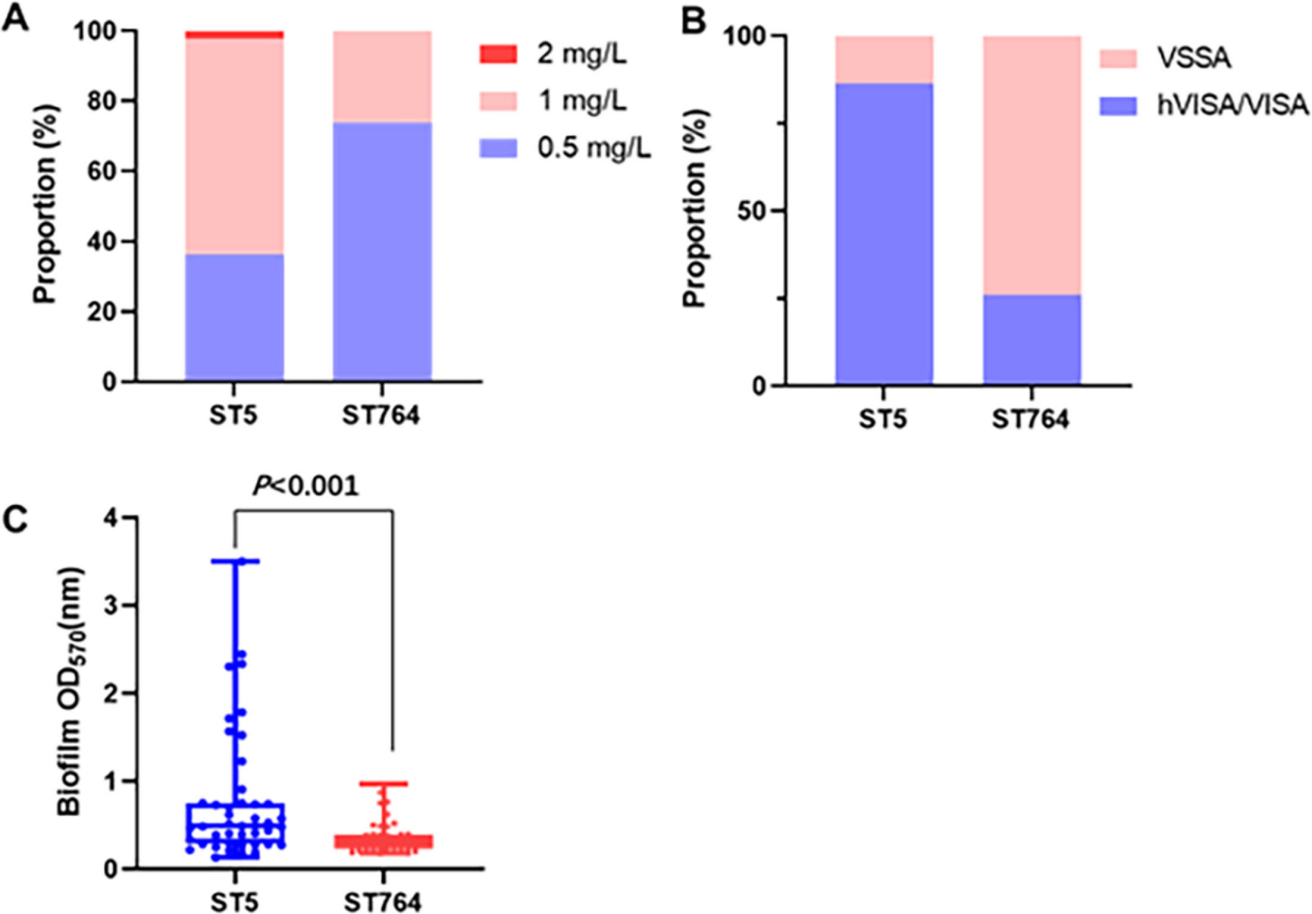
Vancomycin minimum inhibitory concentration (MIC) distribution (**A**), hVISA/VISA detection rate (**B**), and biofilm expression (**C**) of ST5-MRSA and ST764-MRSA.

To assess the distinctions between ST5 and ST764 bacterial phenotypes, we conducted vancomycin MIC assay, heterogeneous vancomycin-intermediate *S. aureus* (hVISA) screening, and biofilm assessment on all the clinical strains. ST764-MRSA and ST5-MRSA strains showed the same MIC_90_ value but lower vancomycin MIC_50_ than that in the ST5-MRSA strain (0.5 mg/L vs 1 mg/L) (Fig. 2A). Similarly, ST5-MRSA strains had a higher detection rate of VISA and hVISA (86.4% vs 26.1%, *P*<0.001) (Fig. 2B) and increased expression of biofilm (OD_570nm_: 0.491 vs 0.304, *P* < 0.001) in comparison to the ST764 strains (Fig. 2C).

Furthermore, molecular typing of ST5-MRSA and ST764-MRSA was also investigated to fully explore their distinctions and potential implications for vancomycin treatment and corresponding clinical outcomes (Table 3). Both ST5-MRSA and ST764-MRSA isolates were typed as SCC*mec*II and accessory gene regulator II (*agr*II). In terms of the *S. aureus* protein A (*spa*) typing, ST764-MRSA are highly clustered in t002 (93.5%), while ST5-MRSA were almost equally scattered among t311 (14/44, 31.8%), t2460 (14/40, 31.8%), and t002 (13, 29.5%).

**TABLE 3:** Genotypes of ST5-MRSA and ST764-MRSA isolates

Characteristic^*a*^	Total (*n* = 90)	ST5^*b*^ (*n* = 44)	ST764 (*n* = 46)	*P* value^*c*^
Molecular typing				
SCC*mecII*	90 (100%)	44 (100%)	46 (100%)	NA
*agrII*	90 (100%)	44 (100%)	46 (100%)	NA
*spa-*t311	14 (15.6%)	14 (31.8%)	0 (0%)	**<0.001**
*spa*-t002	56 (62.2%)	13 (29.5%)	43 (93.5%)	**<0.001**
Adhesion and toxins				
*clfA*	74 (82.2%)	34 (77.3%)	40 (87.0%)	0.277
*clfB*	75 (83.3%)	35 (79.5%)	40 (87.0%)	0.405
*fnbA*	83 (92.2%)	38 (86.4%)	45 (97.8%)	0.056
*fnbB*	53 (58.9%)	24 (54.5%)	29 (63.0%)	0.521
*sdrC*	79 (87.8%)	38 (86.4%)	41 (89.1%)	0.755
*sdrD*	88 (97.8%)	42 (95.5%)	46 (100%)	0.236
*sdrE*	81 (90.0%)	36 (81.8%)	45 (97.8%)	**0.014**
*cna*	0 (0%)	0 (0%)	0 (0%)	NA
*ebpS*	90 (100%)	44 (100%)	46 (100%)	NA
*lukS-PV*	0 (0%)	0 (0%)	0 (0%)	NA
*lukF-PV*	90 (100%)	44 (100%)	46 (100%)	NA
*hla*	90 (100%)	44 (100%)	46 (100%)	NA
*hlb*	90 (100%)	44 (100%)	46 (100%)	NA
*hld*	90 (100%)	44 (100%)	46 (100%)	NA
*tst*	39 (43.3%)	39 (88.6%)	0 (0%)	**<0.001**
*sea*	11 (12.2%)	11 (25.0%)	0 (0%)	**<0.001**
*seb*	43 (47.8%)	0 (0%)	43 (93.5%)	**<0.001**
*sec*	39 (43.3%)	39 (88.6%)	0 (0%)	**<0.001**
*seh*	0 (0%)	0 (0%)	0 (0%)	NA
*sek*	90 (100%)	44 (100%)	46 (100%)	NA
*sel*	39 (43.3%)	39 (88.6%)	0 (0%)	**<0.001**
*eta*	0 (0%)	0 (0%)	0 (0%)	NA
*etb*	0 (0%)	0 (0%)	0 (0%)	NA
*icaA*	88 (97.8%)	42 (95.5%)	46 (100%)	0.236
*v8*	90 (100%)	44 (100%)	46 (100%)	NA
ACME				
*arcA*	90 (100%)	44 (100%)	46 (100%)	NA
*opp-3c*	0 (0%)	0 (0%)	0 (0%)	NA

^*a*^ ST, sequence type; SCC*mec*, staphylococcal cassette chromosome mec; *agr*, accessory gene regulator; *spa,* staphylococcal protein A; ACME, arginine catabolic mobile element; NA, not applicable.

^*b*^ All variables were summarized as the number of cases (%) in the table.

^*c*^ *P* values< 0.05 are shown in bold.

Adhesion factors facilitate the formation of biofilms, enabling MRSA to evade host defenses and impact its pathogenicity. Among the adhesion genes (*clfA*, *clfB*, *fnbA*, *fnbB*, *sdrC*, *sdrD*, *sdrE*, *ebpS*), the *sdrE* gene was significantly different between the two groups (81.8% vs 97.8%, *P* = 0.014) (Table 3). On the other hand, virulence genes impact infection severity, antibiotic response, and outcomes in CA-MRSA and HA-MRSA. No obvious differences in leukocidin genes (*lukS-PV*, *lukF-PV*) and hemolysin genes (*hla*, *hlb*, *hld*) were identified between ST5-MRSA and ST764-MRSA. Notably, ST764 lacked the superantigen genes *tst*, *sec*, and *sel*, whereas 88.6% (39/44) of ST5 strains expressed these genes; *sea* gene was expressed in 25.0% (11/44) of ST5-MRSA but none in ST764-MRSA (Table 3). On the other way, lacking in ST5, *seb* genes were identified in 93.5% (43/46) ST764-MRSA.

ACME *arcA* was detected in all ST5 and ST764 clinical strains, but no *opp-3c* gene, indicating both ST5-MRSA and ST764-MRSA were ACME-II' type.

### Clinical outcomes and risk factors

**Fig 3:**
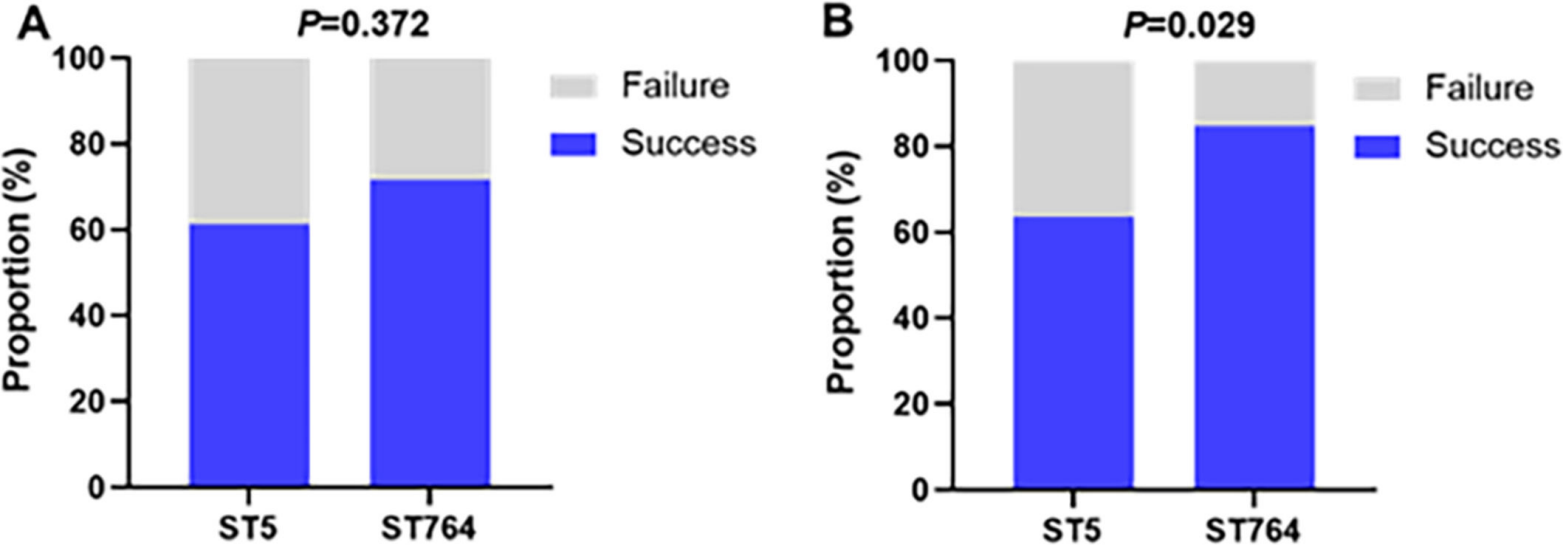
Clinical efficacy (**A**) and bacteriological efficacy (**B**) of vancomycin in the treatment of patients with ST5-MRSA and ST764-MRSA infections.

As for the clinical and bacterial efficacy of vancomycin treatment, the two clonal groups also showed different profiles (Fig. 3). The rate of improvement in clinical signs and symptoms was lower in the ST5-MRSA-infected patients compared with ST764, but not significantly different (61.4% vs 71.7%, *P* = 0.372). Bacterial eradication was also lower in ST5-MRSA patients with statistical significance against ST764 patients (63.6% vs 84.8%, *P* = 0.029).

**Fig 4:**
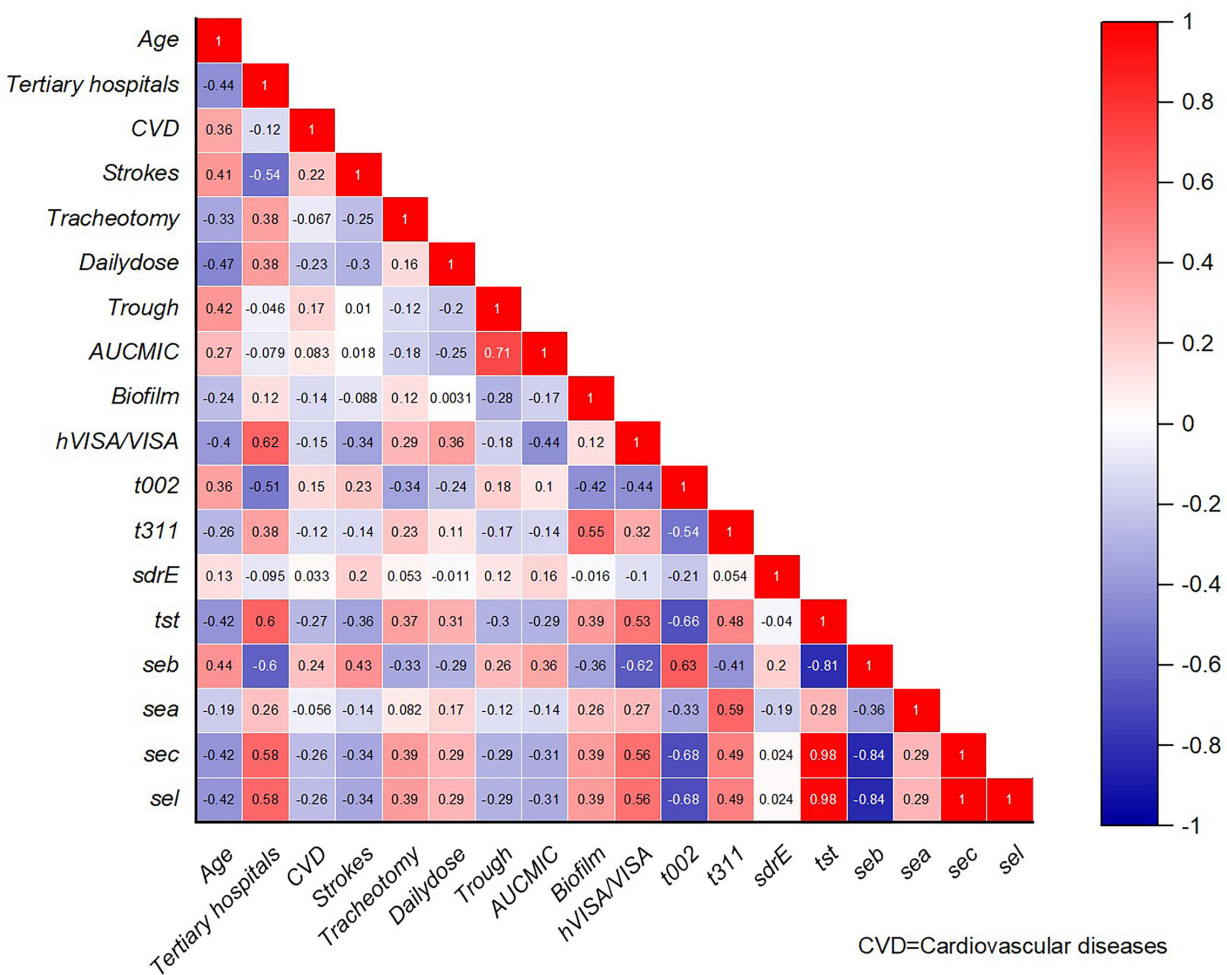
Correlation analysis of variance variables.

All significant variables identified from patient features, vancomycin PK/PD indices, and MRSA strain phenotype and genotype comparison were included in the correlation analysis (Fig. 4). *C*_min_ was found to be positively correlated with AUC_0-24_/MIC, and the *tst* gene was negatively correlated with *seb* and positively correlated with hVISA/VISA phenotype. The prevalence of the *tst* gene was 68.0% (34/50) in hVISA/VISA strains and 12.5% (5/40) in VSSA strains. Subsequent multivariate analysis (Table 4) including all selected significant variables showed that the *tst* toxin was an outstanding risk factor for bacterial persistence with vancomycin [adjusted odds ratio (aOR), 4.509; 95% confidence interval (CI), 1.216 to 16.724, *P* = 0.024]. The Hosmer-Lemeshow goodness-of-fit test revealed a satisfactory model fit, with a *P* value of 0.575.

**TABLE 4:** Multivariate logistic regression of risk factors associated with bacterial persistence in patients with ST5-/ST764-MRSA infection after vancomycin treatment

Risk factor^*a*^	Univariate analysis result OR (95% CI)	Multivariate analysis result	
		aOR (95% CI)	*P* value^*b*^
Age, years	1.009 (0.984, 1.035)	1.003 (0.970, 1.038)	0.860
Tracheotomy	1.700 (0.610, 4.741)	1.058 (0.323, 3.465)	0.926
Vancomycin daily dose	0.910 (0.417, 1.985)	0.824 (0.296, 2.300)	0.312
AUC_0-24_/MIC	1.000 (0.999, 1.002)	1.000 (0.999, 1.001)	0.952
Biofilm	0.709 (0.329, 1.525)	1.044 (0.435, 2.507)	0.923
*sdrE*	1.225 (0.236, 6.367)	1.132 (0.179, 7.159)	0.895
*tst*	**3.359 (1.245, 9.066**)	**4.509 (1.216, 16.724**)	**0.024**
*sea*	0.614 (0.123, 3.075)	0.341 (0.061, 1.916)	0.222

^*a*^ ST, sequence type; AUC_0-24_/MIC, 24-hour area under the curve over minimal inhibitory concentration; OR, odds ratio; CI, confidence interval; aOR, adjusted odds ratio.

^*b*^ *P* values< 0.05 are shown in bold.

## DISCUSSION

This study assessed the efficacy of vancomycin treatment against both ST5-MRSA and ST764-MRSA infections, finding that bacterial eradication was more difficult in the case of ST5-MRSA compared to ST764-MRSA. Between ST5-MRSA and ST764-MRSA infections, clinical characteristics differed in age, hospital setting, stroke, and tracheotomy occurrence for the two patient groups. Patients with ST764-MRSA were older compared to those with ST5-MRSA (77 vs 61 years, *P* < 0.001), which may be partially attributed to the fact that ST764/*SCCmec*II-MRSA carries features of CA-MRSA and is therefore more likely to spread, especially among older patients in the community and hospital settings. Baker et al. (17) investigated the risk factors for CA-MRSA infections in two hospitals in New York from 2006 to 2012, and multifactorial analysis revealed age ≥65 years (OR 2–3, 95% CI: 1.2–4.5) as one of the risk factors. In our study, ST5-MRSA mainly originated from tertiary hospitals, while ST764 was sourced from secondary regional hospitals. Previous studies, such as Peng et al. (18), similarly indicated a higher proportion of HA-MRSA in tertiary hospitals, with 660 HA-MRSA and 175 CA-MRSA strains among 835 MRSA isolates. Additionally, patients in the ST5-MRSA-infected group had higher rates of tracheotomy, possibly because these patients were more likely to develop hospital-acquired infections, similarly revealed in a retrospective case-control study that invasive medical procedures, including tracheal intubation and tracheotomy, were risk factors for hospital-acquired pneumonia (*P* < 0.001) (19).

In terms of bacterial susceptibility, the vancomycin MIC_90_ was 1 mg/L for both ST5 and ST764 strains. However, the MIC_50_ was 1 mg/L and 0.5 mg/L, respectively. Interestingly, the hVISA/VISA detection rate in ST5-MRSA isolates was significantly higher compared to ST764-MRSA, which has not been reported in previous studies. These findings suggest that ST764-MRSA exhibited greater sensitivity to vancomycin than ST5-MRSA. Biofilm expression of ST5-MRSA isolates was also significantly higher than ST764-MRSA (0.491 vs 0.304, *P* < 0.001), which is similar to those of Suzuki et al. (20).

The presence of an arginine deaminase system coding for ACME is one of the features of CA-MRSA, which carries *arc* and *opp-3* regions in the classical USA300 (CC8 CA-MRSA) and has a potential role in enhancing the ability of pathogenic bacteria to grow and survive in the host (21). Both ST5-MRSA and ST764-MRSA in this study were ACME-II' type, carrying only the *arcA* gene and lacking the *opp-3c* gene. This is in line with Takano et al. (8) who first isolated and characterized seven strains of ST764/SCC*mec*II/t002, which carried the *arcA* gene and lacked the *opp-3c* gene, from community and hospital-acquired patients in Japan; however, the other seven strains of ST5/SCC*mec*II/t002 did not carry the aforementioned gene. Urushibara et al. (14) and Kawaguchiya et al. (22) found expression of *arcA* in ST5-MRSA, suggesting that the classical ST5 New York/Japan clone strain may also enhance growth ability in the host by acquiring ACME-II'.

Multivariate analysis suggests that virulence factors, such as the *tst* gene, may be an important risk factor responsible for differential vancomycin efficacy between ST5-MRSA and ST764-MRSA infections. Although *tst*, *sea*, *seb*, *sec*, and *sel* are all superantigen genes, *seb* is expressed in ST764-MRSA but not in ST5-MRSA. *seb* is thought to act as an immune evader during staphylococcal infections (23), thereby promoting infection disseminated in the community. For example, Xie et al. (24) found that the *seb* gene was detected in 52.2% of CA-MRSA isolates, but not in HA-MRSA, speculating that the *seb* gene may be a marker of CA-MRSA isolates. On the other hand, Ho et al. (25) detected that most of the ST5-SCC*mec*II isolates carried both *sec* and *tst* genes, but not in the ST239-SCC*mec*III isolates of HA-MRSA. This suggests that *tst* and *sec* may be ST5-specific features, rather than characteristic genes of all HA-MRSA strains. Additionally, the absence of the *pvl* gene, a characteristic of CA-MRSA, was consistent with the literature (8) in the ST764-MRSA strains.

The difference in efficacy between ST5-MRSA and ST764-MRSA may also be related to the difference in vancomycin trough concentration and AUC_0-24_/MIC between the two groups. The 2020 vancomycin international consensus guidelines recommend maintaining vancomycin AUC_0-24_/MIC at 400–600 (15, 16). The 2020 Chinese vancomycin TDM guidelines also state that for adult patients with MRSA infection, vancomycin trough concentrations should be kept at 10–20 mg/L (26). In our analysis, the median vancomycin trough concentration in ST5-MRSA infection patients was 10.09 mg/L and 14.37 mg/L in the ST764-MRSA group. The median AUC_0-24_ was around 400 mg·h/L in both groups, although AUC_0-24_/MIC was within the recommended threshold, the differences were significant (500 vs 732, *P* < 0.001), indicating the MIC values contribute to the parameter difference. Multifactorial analysis showed that the genetic characteristics of ST5-MRSA and ST764-MRSA are risk factors for bacteriological failure after vancomycin treatment, warranting different vancomycin treatment strategies might be recommended for ST5-MRSA and ST764-MRSA.

Limitations of this study include the absence of *psmα*, *hld*, or ACME *arcA* expression measurements in ST764 and ST5. Further investigation of these virulence factors within our study is warranted.

In summary, there were significant differences in the characteristics of patients and pathogens between ST5-MRSA and ST764-MRSA infections, likely due to the fact that ST764-MRSA, while a variant of ST5-MRSA, exhibits features of CA-MRSA. Patients with ST5-MRSA infections had a lower bacterial eradication rate than those with ST764-MRSA infections. Moreover, virulence factors such as *tst* were found to be the risk factors for treatment failure in ST5 infections. These findings suggest the need for personalized vancomycin treatment strategies based on the genetic characteristics of MRSA isolates. Further studies are needed to confirm these results.

## MATERIALS AND METHODS

### Study design and bacterial isolates

This observational study was conducted at five hospitals from July 2012 to June 2020, using a database constituted of two prospective multicenter clinical studies that were approved by the Ethics Committee of Huashan Hospital and registered with the China Clinical Trials Registry (ChiCTR-OPC-16007920 and ChiCTR-OPC-17012567).

All clinical strains of *S. aureus* prior to vancomycin administration were collected and identical strains from the same patient were excluded. For patients diagnosed with pneumonia, eligible sputum specimens were defined as those with low magnification observation of squamous epithelial cells ≤10 and a minimum leukocyte count of ≥25. MIC of oxacillin and vancomycin were determined by agar dilution method at a CHINET microbiology laboratory (27). Oxacillin ≥ 4 mg/L was defined as MRSA.

### Genotyping

The MRSA strains were extracted using the TaKaRa MiniBEST Bacteria Genomic DNA Extraction Kit Ver.3.0 (Takara Biomedical Technology Co., Ltd., Beijing, China) and stored at −70°C before sequencing. A 300-bp paired-end library was constructed using the NEXTflex DNA Sequencing Kit (Bio Scientific, AZ, USA), and 2 × 150-bp paired-end sequencing was performed on the Illumina X10 platform (Illumina, San Diego, CA, USA). Genome assembly was conducted using the Velvet 1.0.15 program (28), with various hash lengths and coverage cutoffs. Sequence data of seven housekeeping genes (*arcC*, *aroE*, *glpF*, *gmk*, *pta*, *tpi*, *yqiL*) were extracted and analyzed for multifocal sequence typing (MLST) according to the pubMLST database (https://pubmlst.org/). Only ST5-MRSA and ST764-MRSA strains were included in this study.

The molecular typing results of Takano et al. (8) showed that ST764 and ST5-MRSA were both *SCCmec*II and *agr*II. In this study, SCC*mec* type II (*kdpC/B/E*, GenBank accession number BA000018.3) and *agr*II (GenBank accession number AF001782) were used as reference for confirmation. The *spa* gene of the bacteria was extracted and classified as *spa* genotypes according to the Ridom SpaServer database (http://www.spaserver.ridom.de/). Virulence and adhesion genes were screened using the Virulence Factor Database (VFDB, http://www.mgc.ac.cn/VFs/main.htm). A total of 25 virulence or adhesion genes, commonly found in *S. aureus* (29), were included in the analysis. These genes were selected based on the factors that may influence vancomycin efficacy (30). ACME was identified by *arcA* (GenBank accession number BA000017.4: c2790552-2789317) and *opp-3c* (GenBank accession number MF346685.1:43104–43871). ACME-II' was defined as carrying only the *arcA* and not the *opp-3c* region (8, 21). BLAST software was used to find the similarity and respective lengths between sequences.

### hVISA screening

As previously described, we used a modified population analysis area under the curve method (PAP-AUC) to screen for hVISA (31, 32). For classification, strains with ratios greater than 1.3 were categorized as VISA, ratios between 0.9 and 1.3 were classified as hVISA, and strains with ratios less than 0.9 were considered as vancomycin-susceptible *S. aureus* (VSSA).

### Biofilm formation

Biofilm formation was determined by a crystalline violet method (32) and controlled against the ATCC29213 standard strain. Fresh overnight bacteria were selected, adjusted for turbidity to 0.5 McFarland, and shaken overnight at 37°C 180 rpm. Bacterial broth was diluted 1:100 and inoculated into BHI + 1% glucose broth after adding to 96-well Costa plates making three replicate wells per strain. After incubation at 37°C for 24 hours, the wells were washed three times with phosphate-buffered saline [(PBS); pH 7.2]. Methanol was used to stabilize the biofilm, followed by 15 minutes of staining with 1% crystal violet dye. The wells were washed with slow-flowing water until the water was colorless and dried at room temperature. Each well was dissolved by adding 0.2 mL of an aqueous 80% ethanol solution, and the optical density (OD) was measured at 570 nm on a spectrophotometer (model ELX800; BioTek, Winooski, VT, USA).

### Patients and clinical data collection

Adult patients (age ≥18 years) with clinical signs, symptoms, laboratory tests, and microbiological culture results diagnosed with ST5-/ST764-MRSA infection and taking vancomycin for ≥5 days were included in the data set. Patients were excluded if they had received any other anti-Gram-positive agents besides vancomycin for ≥24 hours within 72 hours prior to admission, if Gram-positive bacteria colonized, and if they were pregnant or lactating women or if they were taking concomitant aminoglycosides. Patient demographics, ICU admissions, underlying diseases, curative treatment, clinical laboratory tests, bacteriological tests, and vancomycin doses were collected. In this study, we included underlying diseases such as cardiovascular diseases, diabetes, and strokes, as they have the potential to impact the efficacy of anti-infection therapy (32, 33).

### Vancomycin TDM and PK/PD analysis

Serum *C*_min_ samples were collected no earlier than 0.5 hours prior to the fifth dose, and *C*_max_ samples were collected 0.5–1 hour after dosing. If the patient’s creatine clearance (CLCr) was less than 30 mL/min, a serum sample was collected at the second dose. Vancomycin serum concentrations were determined by fluorescence polarization immunoassay (FPIA) or chemiluminescent microparticle immunoassay (CMIA) with a linear range of 3 to 100 mg/L.

A previously published method (32) was used to simulate the concentration-time profile of patients and calculate the PK/PD indices of patients after vancomycin dosing. *C*_min_, *C*_max_, and AUC_0-24_ values were calculated as weighted means using the dose and interval of administration, respectively. AUC_0-24_/MIC was evaluated based on the calculated AUC_0-24_ and laboratory vancomycin MIC values.

### Clinical outcome definitions

Clinical outcomes include clinical efficacy (improvement in clinical signs and symptoms) and bacteriological efficacy (eradication of bacteria). Improvement in clinical signs and symptoms means that the patient’s clinical signs and symptoms of infection and laboratory test results (other than bacteriological tests) have returned to normal or pre-infection status, and vancomycin is no longer required for 7 days since vancomycin discontinuation. The assessment of clinical signs and symptoms may vary depending on the type of infection. For instance, in patients with pneumonia, typical clinical signs and symptoms include fever, cough, sputum production, both dry and moist rales, and observable changes in imaging. Laboratory tests such as white blood cell count, neutrophil count, and serum creatinine values are also considered. Bacterial eradication means that the original pathogen cannot be cultured in 7 days after stopping vancomycin treatment and antibiotics against Gram-positive organisms are no longer required.

### Statistical analysis

All statistical analyses were performed using SPSS 19 (SAS Institute, Cary, NC, USA), and figures were plotted using GraphPad Prism software version 8.4.3 (GraphPad Software, LLC, San Diego, CA, USA). Categorical variables were presented in descriptive statistics as number of cases (percent %) and continuous variables as median [interquartile range (IQR)]. Univariate comparisons were performed using Fisher’s exact test for categorical variables and Mann-Whitney *U* test for continuous variables. *P* values less than 0.05 were considered statistically significant. All significant variables were included in the correlation analysis using the OriginPro software version 2023 (OriginLab Corporation, OriginLab Corporation, MA, USA). Variables with correlation coefficients below 0.5 and deemed potentially relevant to the treatment outcomes were included in the final multivariate analysis. The adequacy of the model fit was assessed using the Hosmer-Lemeshow goodness-of-fit test, with a *P* value exceeding 0.05 indicating a satisfactory model fit.
